# Alcohol control policies in Former Soviet Union countries: A narrative review of three decades of policy changes and their apparent effects

**DOI:** 10.1111/dar.13204

**Published:** 2020-11-05

**Authors:** MARIA NEUFELD, ANASTACIA BOBROVA, KAIRAT DAVLETOV, MINDAUGAS ŠTELEMĖKAS, RELIKA STOPPEL, CARINA FERREIRA-BORGES, JOÃO BREDA, JÜRGEN REHM

**Affiliations:** 1 WHO European Office for Prevention and Control of Noncommunicable Diseases, Moscow, Russia; 2 Institute for Clinical Psychology and Psychotherapy, TU Dresden, Dresden, Germany; 3 Institute for Mental Health Policy Research, Centre for Addiction and Mental Health, Toronto, Canada; 4 Institute of Economics, National Academy of Sciences, Minsk, Belarus; 5 Health Research Institute, Faculty of Medicine, Al-Farabi Kazakh National University Almaty, Almaty, Kazakhstan; 6 Health Research Institute, Faculty of Public Health, Lithuanian University of Health Sciences, Kaunas, Lithuania; 7 Department of Preventive Medicine, Faculty of Public Health, Lithuanian University of Health Sciences, Kaunas, Lithuania; 8 Department of Economics, University of Potsdam, Potsdam, Germany; 9 Center for Interdisciplinary Addiction Research, Department of Psychiatry and Psychotherapy, University Medical Center Hamburg-Eppendorf, Hamburg, Germany; 10 Campbell Family Mental Health Research Institute, Centre for Addiction and Mental Health, Toronto, Canada; 11 Department of Psychiatry, University of Toronto, Toronto, Canada; 12 Dalla Lana School of Public Health, University of Toronto, Toronto, Canada; 13 Department of International Health Projects, Institute for Leadership and Health Management, Sechenov First Moscow State Medical University (Sechenov University), Moscow, Russia

**Keywords:** alcohol, alcohol policy, Eastern Europe, Former Soviet Union, mortality

## Abstract

**Issues.:**

The last Soviet anti-alcohol campaign of 1985 resulted in considerably reduced alcohol consumption and saved thousands of lives. But once the campaign’s policies were abandoned and the Soviet alcohol monopoly broken up, a steep rise in mortality was observed in many of the newly formed successor countries, although some kept their monopolies. Almost 30 years after the campaign’s end, the region faces diverse challenges in relation to alcohol.

**Approach.:**

The present narrative review sheds light on recent drinking trends and alcohol policy developments in the 15 Former Soviet Union (FSU) countries, highlighting the most important setbacks, achievements and best practices. Vignettes of alcohol control policies in Belarus, Estonia, Kazakhstan, Lithuania and Uzbekistan are presented to illustrate the recent developments.

**Key Findings.:**

Over the past decade, drinking levels have declined in almost all FSU countries, paralleled by the introduction of various alcohol-control measures. The so-called three ‘best buys’ put forward by the World Health Organization to reduce alcohol-attributable burden (taxation and other measures to increase price, restrictions on alcohol availability and marketing) are relatively well implemented across the countries.

**Implications.:**

In recent years, evidence-based alcohol policies have been actively implemented as a response to the enormous alcohol-attributable burden in many of the countries, although there is big variance across and within different jurisdictions.

**Conclusion.:**

Strong declines in alcohol consumption were observed in the 15 FSU countries, which have introduced various alcohol control measures in recent years, resulting in a reduction of alcohol consumption in the World Health Organization European region overall.

## Background

A large and growing body of research with increasingly refined methodology documents the causality between different dimensions of alcohol consumption and different mortality and morbidity outcomes [1,2]. Patterns of heavy episodic drinking have been shown to increase the health harms over and above level of drinking for some disease outcomes, for example, for ischaemic disease [3]. This implies that a reduction of prevalence of heavy episodic drinking—commonly defined by the World Health Organization (WHO) as a drinking pattern including an intake of at least 60 g of pure alcohol on one occasion within the past 30 days—would lead not only to a reduced individual risk but also to a notable reduction of all-cause mortality at the population level [4,5].

While most of this evidence is based on longitudinal epidemiological studies, some of the strongest evidence for causality between alcohol exposure and health outcomes stems from so-called natural experiments, often with policy or other interventions on alcohol exposure, and then measuring the health outcomes. Although alternative explanations cannot be ruled out in the absence of a random assignment to condition, studying the relation of health outcomes to such policy changes can still provide important insights into the causal direction of the relationship between changing conditions and resulting outcomes, unlike trend analyses that yield only associations and correlations. Such natural experiments involving changes in the availability or affordability of alcohol or any other relevant aspects of exposure to alcohol are probably the only feasible study designs to explore the impact of interventions on alcohol-related harm on a population level.

Globally, a series of historic events demonstrate the stable link between alcohol exposure and all-cause mortality. At the beginning of the 20th century 13 countries, including Norway, Finland, Iceland, Russia, Canada and the USA, instituted national prohibition legislation [6], triggered by strong temperance movements, and the measures adopted had effects on levels of consumption and harm [7–10]. Other historic examples of abrupt declines in alcohol affordability or availability have led to documented declines in alcohol consumption and mortality as well—for instance, the substantial tax increase on distilled spirits in Denmark during World War I and the wine seizures in France during the German occupation of World War II [4]. A more recent example of a large-scale policy change is the last Soviet anti-alcohol campaign of 1985, which was able to reduce alcohol consumption in the world’s largest country and to prevent thousands of deaths according to different estimates [11–15]. The experiment was abandoned prior to the breakup of the Soviet Union [16], and political and popular reactions against it facilitated the opening of alcohol markets and substantial increases in alcohol consumption in the 1990s. After the dissolution of the Soviet Union, various countries experienced a steep rise in mortality and drops in fertility rates and life expectancy. Economic transition and unemployment surges due to the disruption of the labour market, temporary collapses of local health-care systems, increases in alcohol consumption and smoking because of loosened availability and pricing regulations as well as illegal production and smuggling, unhealthy diets and overall lifestyles, combined with psychological stress were featured as the most common explanations for the post-Soviet demographic crisis [17–20].

Most of the studies that exist on the topic document the effects of the 1985 campaign and its repercussions in the succeeding period in the Russian Federation [19,21–25]. The Russian mortality crisis of the 1990s, the role of levels of alcohol consumption and prevalence of heavy episodic drinking, pointing to the importance of alcohol control measures to reverse the worrisome trends, remains the best researched area within this field [26–31]. Research on alcohol policy and its impact on mortality and population health in other former Soviet republics remains scarce, with some notable exceptions for the Baltic States, Belarus, Moldova and Ukraine [32–39]. Most of these studies demonstrate that after the end of the Gorbachev-era interventions, and with independence and more market influences, levels of consumption increased. But, in reaction to the associated harm, some of these states introduced new alcohol policy measures.

Studies that explore recent developments in alcohol control and their effects in countries of Central Asia and the Caucasus are almost non-existent, and what information exists is either outdated or limited in scope [40–42], although available data from the WHO suggests that these countries have stricter alcohol laws than the rest of the region [43]. Many of these restrictions were introduced quite recently, and their effects have not yet been evaluated.

The present contribution aims to provide a general overview of the alcohol policy landscape in the 15 Former Soviet Union (FSU) countries (Armenia, Azerbaijan, Belarus, Estonia, Georgia, Kazakhstan, Kyrgyzstan, Latvia, Lithuania, Moldova, Russia, Tajikistan, Turkmenistan, Ukraine and Uzbekistan, for more background information see Table S1, Supporting Information), focusing on the state of implementation of what the WHO has termed the three ‘best buys’ for reducing alcohol-attributable harm: relatively high alcohol taxes or other state interventions to increase the price of alcoholic beverages; limits on the timing, locations or other aspects of alcohol availability; and restrictions on advertising, marketing and other promotional activities. These are interventions that have been found to be highly effective and cost-effective, feasible and appropriate to implement even where resources are limited [44,45]. A special focus of this paper’s policy mapping is on the documentation of changes in alcohol policies in countries where no, or very little, literature is available, such as the Central Asian republics. Selected country cases are discussed in country snapshots, highlighting achievements and setbacks in alcohol control efforts; these summaries can be used as entry points for further impact analyses. Moreover, the review highlights the long-term trends in levels of alcohol consumption in the 15 FSU countries and trends in all-cause mortality. As is customary, levels of alcohol consumption in a country will be measured as adult alcohol *per capita* (APC) consumption [i.e. litres of ethanol (pure alcohol) divided by the number of inhabitants aged 15 and above [46]].

## Methods

Since the 15 FSU countries are very diverse in their history and present developments, different groupings are used in this article to denote the different sub-groups. First, a geographical approach is used when discussing drinking and mortality trends, grouping the 15 countries into the Eastern European, Transcaucasian and Central Asian country groups. These sub-regions indicate not only the geographical location of the specific countries, but the countries in each group are also somewhat similar in terms of their income level, proportion of Muslim population and APC intake (see Table S1, Supporting Information).

Secondly, political groupings are also used when describing policy implementation across the 15 countries, as alcohol control largely depends on the overall national and international legislative frameworks. After the dissolution of the Soviet Union, the Commonwealth of the Independent States (CIS) was formed in 1991 with 11 countries (Armenia, Azerbaijan, Belarus, Kazakhstan, Kyrgyzstan, Moldova, Russia, Tajikistan, Turkmenistan, Ukraine and Uzbekistan) agreeing and signing the founding Alma-Ata Protocols, while the three Baltic States (Estonia, Latvia and Lithuania) did not sign the declaration, and Georgia did not participate. The three Baltic countries joined the European Union in 2004, while Turkmenistan became an Associative Member of the CIS and Ukraine ended its participation in May 2018. Also, in 2014 a treaty of the Eurasian Economic Union (EAEU) was signed, which now consists of Armenia, Belarus, Kazakhstan, Kyrgyzstan and Russia. The EAEU is aimed at greater economic integration between these countries, with harmonised common markets for goods, services, energy, agriculture and other economic sectors (for an overview of political unions, see Figure S1, Supporting Information). As part of the EAEU agreements, the Member States are free to establish their own minimum prices on alcoholic beverages with ethanol content (alcohol by volume; ABV) of 28% and above and raw ethanol. They are also obliged to harmonise their alcohol excise rates at a certain level every 5 years to prevent large differences between national alcohol prices and thus avoid stimulating cross-border shopping and smuggling.

The present study relies on a multi-methods approach in a narrative review of available policy databases, literature and legislative acts, as well as WHO country-level estimates on trends in alcohol consumption.

For the general overview of the implementation of the ‘best buys’, data from the WHO Global Survey on Alcohol and Health from 2016 were used as the initial information source [43], and the policy mapping was based on a set of core indicators that were reported for 30 European countries in 2018 [47]. Indicators were updated for 2020 through a hand-search of relevant legislative documents and scientific literature in the field, using national government websites and legal counselling online platforms (see Table S2, Supporting Information, for a list of sources). For 11 countries, legislative documents could be reviewed within the ‘Legislation of the CIS countries’ database, which contains laws and other regulatory documents of the nine CIS countries as well as Ukraine and Turkmenistan [48].

Data on adult APC consumption of a country were taken from the WHO Global Survey on Alcohol and Health and the 2019 Global Survey on Progress on Sustainable Development Goal Health Target 3.5[49], and calculated as 3-year moving averages for total APC consumption, consisting of recorded and unrecorded alcohol use and adjusted for tourist consumption [46,50]. Age-standardised rates for all-cause mortality were retrieved from the Global Health Data Exchange Databank [51]. Data on country classification by income level and purchasing power parities were obtained from the World Bank [52].

Additionally, consultations with experts and stake-holders from selected countries were carried out for data updates and validation and interpretation of results. This part of the analysis was also informed by a workshop on alcohol control policies in the CIS countries that took place in December 2019 in Moscow, where delegates from all the Member States of the CIS provided input on the current state of alcohol policy implementation in their countries [53].

## Results

### Trends in alcohol consumption and all-cause mortality

**Figure 1.**
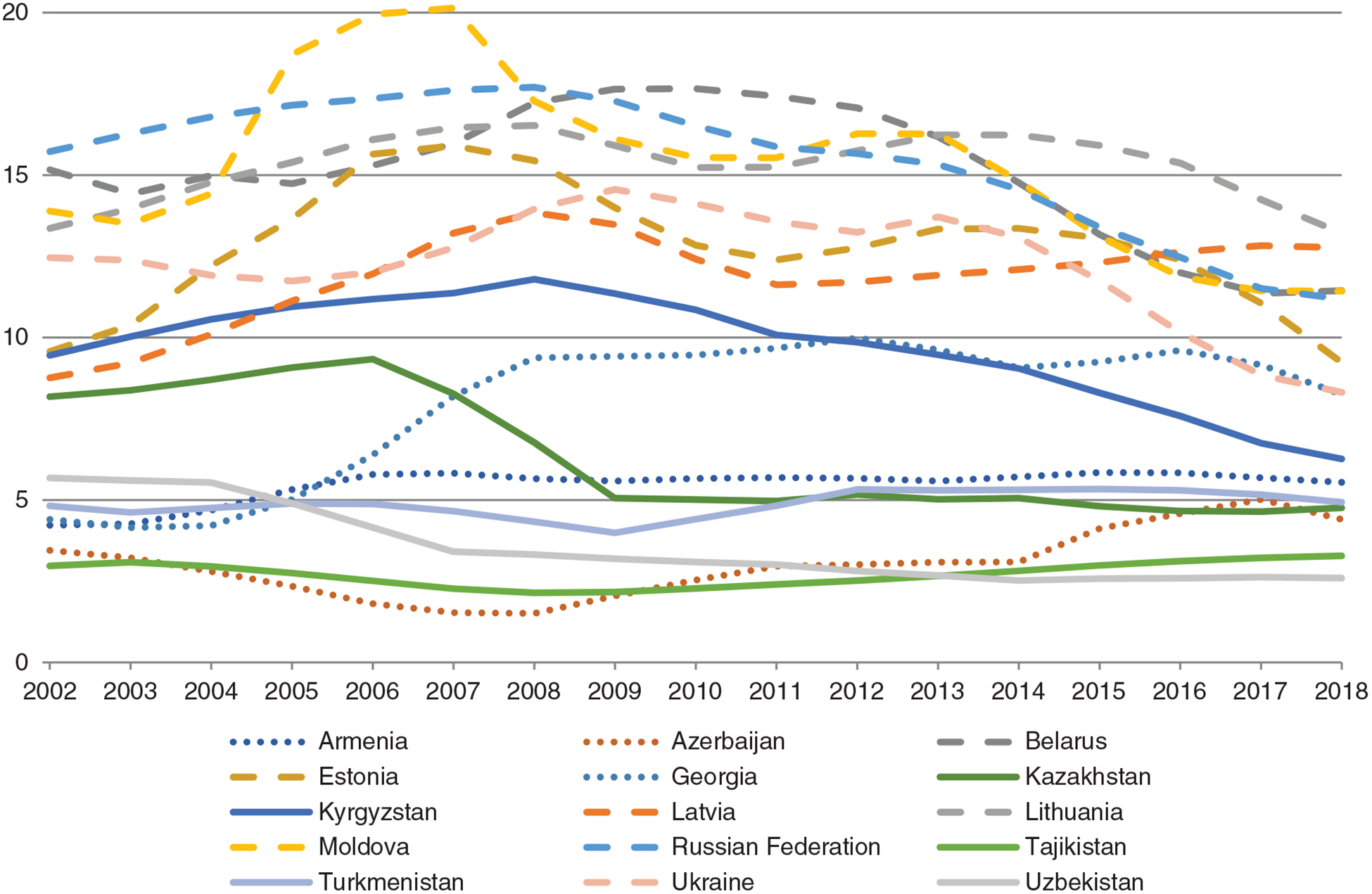
Trends in total alcohol per capita consumption (15+) in litres of pure alcohol, 3-year moving averages. Total consumption includes recorded and unrecorded alcohol use and is adjusted for tourist consumption. Dashed lines: countries of Eastern Europe; dotted line: countries of Transcaucasia; full lines: countries of Central Asia. Source: WHO Global Survey on Alcohol and Health and the 2019 Global Survey on Progress on Sustainable Development Goal Health Target 3.5 [49].

The 15 FSU countries vary greatly in terms of their APC consumption, and most of them have shown large fluctuations of drinking levels over the past two decades (see Figure 1).

Countries with the highest APC intake and largest fluctuations are located in Eastern Europe and are also the ones where the largest relative declines in drinking were observed. Drinking levels in the Transcaucasian countries of Armenia, Azerbaijan and Georgia are much lower, though a substantial APC increase occurred in Georgia and Azerbaijan over the past 15 years. Drinking trends and levels of the five Central Asian countries are more diverse, although they seem to converge over time. While Kazakhstan and Kyrgyzstan had a relatively high alcohol intake as compared to the rest at the beginning of the 2000s, both countries have decreased their consumption substantially, while drinking levels remained stable or increased in the other three countries.

It is worth noting that while the largest relative decreases in APC between 2010 and 2018 occurred in Eastern European countries (Belarus, Estonia, Russia and Ukraine), the largest relative increases appeared in countries with a relatively large Muslim population, namely Azerbaijan, Tajikistan and Turkmenistan (see Table S1, Supporting Information). The only exception to this pattern was Kyrgyzstan, where a strong decline was observed in the same time period.

**Figure 2.**
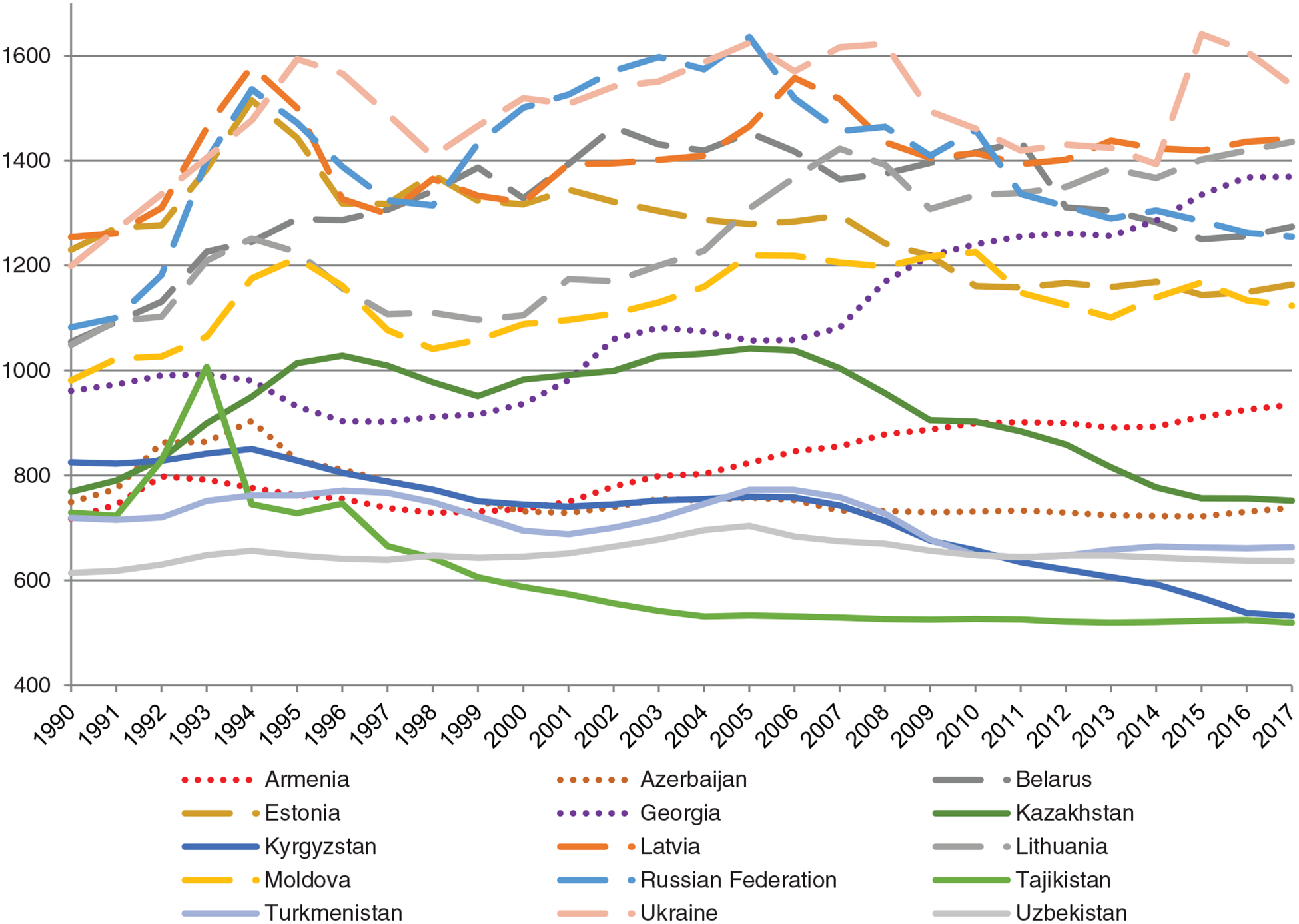
Age-standardised mortality rates per 100 000 population for all causes of mortality. Source: Global Health Data Exchange Databank [51].

When looking at the trends in all-cause mortality in the 15 FSU countries for the period 1990–2017, a similar pattern emerges (Figure 2). Overall, Eastern European countries demonstrate higher mortality.

Eastern European countries have also demonstrated more sizable mortality fluctuations than others, and a general decline over the recent period, while mortality rates in Central Asia have generally been lower but have also declined over time. In the Transcaucasian countries, mortality rates rose steeply for Georgia and moderately for Armenia and remained somewhat stable for Azerbaijan. In all Eastern European countries (with the exception of Belarus) and Kazakhstan a distinct mortality peak is observed in the 1990s and a subsequent rise and fall in the 2000s. This reflects the post-Soviet demographic crisis these countries were facing at that time (see above for more explanations and references). Belarus is the only country without such a pronounced peak; mortality there steadily increased until the mid-2000s, but has fallen since then. Tajikistan demonstrates a steep rise in mortality between 1991 and 1993, which likely reflects the lives lost in the first years of the Tajikistani Civil War, and a steady decline ever since. Out of all countries, Tajikistan has the lowest mortality rates, while Ukraine has the highest. The rate in Ukraine has increased in recent years, which is likely the result of the armed conflict happening in the eastern part of the country.

### Availability of alcohol—tightening restrictions in various FSU countries

For a detailed overview of the main indicators of three ‘best buys’, namely alcohol availability, pricing and marketing in the region, see Table 1.

State-owned monopolies are considered to be the most effective structural arrangement for the regulation of alcohol availability, followed by licensing systems that dictate the exact conditions of sale [5]. After the dissolution of the Soviet Union and its state monopoly on alcohol production and sale, most FSU countries have moved to a licensing system for production, import, export and sale of alcoholic beverages. None of the countries has maintained a retail monopoly. However, out of the 15 FSU countries, a total of three—Belarus, Moldova and Turkmenistan—have preserved a government monopoly on the production of alcohol.

Still, Armenia, Georgia and Estonia do not have active licensing systems for retail sale of all alcoholic beverages and rely on self-registration of alcohol sellers, and Belarus and Moldova do not have a licensing system for retail sale of beer, despite the existence of state monopolies on production.

Increasing the minimum legal drinking age is another effective measure to regulate alcohol availability. Overall, the legal drinking age in the region is relatively high compared to countries of the European Union, ranging from 18 years in most of the countries to 20 years in Lithuania and Uzbekistan and to 21 years in Kazakhstan. Kazakhstan is therefore one of the few countries worldwide that has such a high minimum legal age implemented at the national level.

The introduction of a higher minimum drinking age and longer restrictions on hours of sale in Lithuania is a relatively new development, as these interventions were introduced as part of a whole ‘best buy’ package in response to the world’s highest alcohol consumption levels in 2014–2018 (see Box 1).

As for restrictions of alcohol availability, seven out of 15 countries restrict sales of alcoholic beverages at petrol stations and have national restrictions on hours of off-premise sales. Only Uzbekistan has partial restrictions on hours of on-premise sales and service. A special case of time restrictions can be found in Kazakhstan, where off-premises sale hours depend on alcohol content: sales of alcoholic beverages with an ABV of 30% and below are prohibited between 11 pm and 8 am, whereas spirits with an ABV >30% cannot be sold between 9 pm and 12 pm of the following day (for more information on alcohol control in Kazakhstan, see Box 2).

Uzbekistan is the only country in the region that has restrictions on sale locations and hours as well as on the density of outlets, although the density restrictions were loosened in recent years. Besides Uzbekistan, no other country restricts the density of outlets.

Along with Ukraine, Uzbekistan has also abolished its state monopoly on alcohol production only recently, moving instead to licensing procedures as softer control mechanisms. Moreover, sales hours vary greatly by outlet, depending on the specific license, so that the availability restrictions are rather ambiguous and difficult to interpret (see Box 3 for more information on Uzbekistan’s alcohol control changes).

Considering the recent changes in alcohol availability across the entire WHO European region, it is apparent that the strongest new restrictions adopted were in some of the FSU countries [43,54].

### Pricing—the relative success of minimum prices as an additional pricing mechanism

There is a wide variety of pricing schemes across the region, ranging from strict control in the form of government price-fixing to free markets with little or no specific regulation of the alcohol market (for an overview, see Table 1).

Most of the FSU countries tax the production of ethyl alcohol, and six out of 15 countries adjust their alcohol taxes for inflation, at least for some beverage types. This is more than is true for EU countries, where most of the traditional wine-making countries do not have an alcohol excise tax on wine at all, let alone inflation adjustment procedures [55].

One of the most interesting examples of strict government control over production quotas, fixed alcohol prices and differential pricing schemes might be Belarus, where comprehensive measures through centralised state control have been shown to have decreased alcohol consumption (Box 3). Although very few formal analyses of the situation in Belarus exist so far, a recent study highlights the key role of the state-run alcohol production monopoly in forming the alcohol market and emphasises the responsibility of the government in protecting the public’s health, including from the state’s own interest in profit and revenue from alcohol sales [32,56].

As for other pricing mechanisms beyond taxation, a total of eight countries have a minimum retail price for vodka, and four of these countries have an additional minimum price for other beverages, while Armenia has an universal minimum unit price (covering all alcoholic drinks; for an overview, see Table 2).

However, the implementation modes of the minimum prices vary greatly across these countries. The majority have an established minimum price for vodka per litre of final product sold in retail sale, but there are minimum prices for some other forms as well. For instance, Armenia has a specific minimum unit price of 6000 Armenian Drams per litre of pure alcohol [57] for all alcoholic beverages. Ukraine has a similar specific minimum unit price for each of a wide range of alcoholic beverages with the exception of beer, as established by the Resolution of the Cabinet of Ministers of Ukraine [58].

Belarus has a joint minimum retail price for vodka, spirits and fortified wines with ABV >28% and a separate minimum price for fortified wines with an ABV of 28% and below [59]. Russia has separate minimum price categories for vodka and other spirits with ABV >28%, as well as cognac, brandy and sparkling wine [60]. At the same time, there is a gradation of minimum prices for vodka and spirits, depending on their ABV, and there are different minimum prices for retail and wholesale vendors as well as distributors [61]. Belarus and Russia also have distinct minimum wholesale prices for raw ethanol that is used for the alcoholic beverages. Moreover, Russia has a specific decree in place that forbids the sale of non-beverage alcoholic products with an ABV >28% at a lower price than the established minimum retail price for vodka and spirits, to discourage their misuse as surrogates [62].

Kyrgyzstan has a minimum retail price for nationally produced vodka [63]. Uzbekistan has retail and wholesale minimum prices for wine, vodka and cognac [64]. Moldova introduced a minimum price on vodka in 2010 but, although it was never officially repealed, it is de facto inactive, as the average vodka prices are now much higher than the established floor price because of the inflation of the value of the national currency [65].

It is worth noting that minimum prices have been introduced in all the Member States of the EAEU and the treaty of the union explicitly states that Member States can do so.

At a price equivalent to more than 25 international dollars (I$) per litre, Ukraine has by far the highest minimum price for vodka in 2020, followed by Belarus and Russia, while Uzbekistan has the lowest. Similarly, Ukraine has currently the highest minimum prices for other alcoholic beverages, while Uzbekistan the lowest. So far, there are no evaluation studies of the effects of minimum price-setting in these countries, although countries like Belarus and Russia had introduced minimum prices on vodka already 20 years ago [26,32]. However, in the case of Russia, for instance, the initial minimum price for vodka was initially at such a low level that it was not noticeable until 2010, when excise rates and minimum prices were systematically increased as part of a long-term strategy to reduce alcohol consumption and harm [66].

### Marketing—implementation of total marketing bans across different media and platforms

In considering alcohol marketing restrictions, we focus here mainly on regulation of marketing through new digital media—the Internet and social media—as well as of promotions through special offers and sales and sponsorship of sports and news events (see Table 1). These areas have become increasingly important platforms for alcohol advertising, especially given that various FSU countries already had full or partial marketing bans across traditional media in place [43].

A total of six countries have a ban on alcohol discount promotion to prevent sales outlets from advertising and offering cheaper deals on alcohol to customers who buy in bulk. Four countries, all in Central Asia (Kazakhstan, Tajikistan, Turkmenistan and Uzbekistan), have a ban on promotion of alcoholic beverages at the point of sale. In addition, some countries have partial restrictions in this regard. Estonia, for instance, introduced restrictions on general visibility of alcoholic beverages at the point of sale in order to decrease impulse purchases (for more information see Box 4).

Seven FSU countries (among them, all the Central Asian countries) have a digital marketing ban in place, prohibiting alcohol advertising on the Internet and social media. Also, six countries ban sponsorship of sports and youth events by alcohol producers or distributors, although they are only partial in the case of two countries; Belarus does not have a ban on sponsorship for youth and sports events for beer, while Russia has a ban on sponsorship of sports events, but not of youth events.

However, six countries—Armenia, Azerbaijan, Georgia, Latvia, Moldova and Ukraine—do not have a ban on any of the reviewed areas, although there are partial restrictions in place in Azerbaijan. Kazakhstan, Turkmenistan and Uzbekistan, on the other hand, have full marketing bans across all areas and media types, while Tajikistan and Russia lack some regulations that the others have, but overall have strong marketing restrictions in place.

Compared to other regions in the world, or the entire WHO European Region, regulations on marketing are rather strict across the FSU countries [67].

## Discussion—FSU countries as under-researched success stories of alcohol control

Overall, trends in alcohol consumption and all-cause mortality show similar patterns in the region, which is in line with the existing evidence that alcohol is one of the main contributors to mortality, especially in the countries in Eastern Europe [37,68].

This mapping of policy measures and changes in the 15 FSU countries since the 1990s demonstrates that there is large variation between them in policy implementation, but that WHO’s three ‘best buys’ are much more often implemented in these countries than in the rest of the WHO European region [43,55]. Not surprisingly, these were also the countries where larger relative decreases in alcohol consumption were observed [43,69].

The country snapshots (see the Boxes) showcase some of the unique alcohol provisions that are not implemented anywhere else in the WHO European region or even globally, at least with such rigor. These regulations range from the online tracking systems for alcohol developed in Russia, and now implemented in Kazakhstan and Uzbekistan, to the strict provisions on mandatory health warnings for alcohol containers and sale outlets in Uzbekistan, and the strict marketing and sponsorship bans across different media types and platforms. The existence of state monopolies on alcohol production, distribution and import/export, as well as the existence of established minimum prices and tax adjustment for inflation in several FSU countries, suggest that governmental control over alcohol production and sales remains a high priority in this part of the region.

There are some obvious limitations to our study. First of all, the chosen methodology was a narrative review and policy mapping, which followed a predefined framework, but is in no way as rigorous as a systematic search procedure. Searches of legislative documents and provisions were mostly carried out in Russian as well as some other national languages, but the research team could not cover all the national languages spoken in the 15 FSU countries. Although a substantial portion of legislative documents of these countries is available in Russian, this availability has been declining in recent years. Therefore, there is a possibility that some of the most recent legislative documents from some countries might have been over-looked in the process. Since the review has focused on legislative documents for alcohol measures, some important gaps might exist in their implementation and overall comparability across countries. For instance, the demonstrated variety of minimum prices in the region shows that it is difficult to compare this measure across countries, as different implementation models exist, and in some cases minimum prices might no longer be effective because of inflation. Another important issue is the enforcement of the measures reviewed. Here, another example would be the bans on alcohol marketing and sponsorship. It is well documented that in some countries with such bans, alcohol producers are promoting not their specific products, but their brand names, for instance through advertisement of non-alcoholic beer [26,35].

The documented experiences of policy implementation in FSU countries offer important insights into the relationship between different policy interventions and levels of drinking and alcohol-attributable harm under real-life conditions. Although the analyses performed as part of this contribution remain descriptive, so that a causal conclusion cannot be drawn on the specific effect of the interventions on the drinking and mortality trends in the region, some of the results can be useful for general discussions of the effectiveness of alcohol policies. One of the most promising areas where more studies are needed is evaluation of the effects of minimum prices and of their interaction with unrecorded alcohol. Pricing policies, including minimum prices, can be less effective if unrecorded alcohol represents a large proportion of total alcohol in a country. Unrecorded alcohol is an umbrella term for alcoholic products that are not accounted for in official statistics on taxation, sales and consumption, but are nevertheless consumed as alcoholic beverages [43]. In FSU countries, this type of alcohol is consumed in all the different forms that exist, ranging from home-produced alcoholic beverages to smuggled, undeclared and/or illegally produced alcohol, and to alcohol surrogates, that is alcoholic products that are (at least officially) not intended for human consumption, but are still consumed as beverages to reach intoxication. Compared to the rest of the European region, consumption of this type of alcohol is estimated to be particularly high in FSU countries, accounting for around 15–30% of all alcohol consumed [68,70]. With the exception of some forms of cross-border shopping, unrecorded alcohol is usually the cheapest form of alcohol available, and is thus popular among heavier drinkers and, in particular, heavier drinkers of lower socioeconomic strata [71]. Despite the growing literature in this field, unrecorded alcohol and its regulation remain an understudied phenomenon and more research is needed on the complex links between consumption of certain types of unrecorded alcohol, socio-economic factors and health outcomes in this region. A clear link has been established between certain combinations of lifestyle factors and disproportionate harm in deprived populations, and the existing literature from Eastern European countries highlights how consumption of surrogate alcohol is linked to marginalisation, social deprivation, hazardous drinking patterns, severe forms of alcohol dependence, and poor mental and physical health, including disproportionate mortality risks [72–79]. It is therefore important to keep unrecorded alcohol in perspective when introducing stronger alcohol policies, as consumers might switch to cheaper and more available alcoholic products. For instance, the relatively large alcohol tax increase in Estonia as part of the three ‘best buys’ implementation led to increasing cross-border purchases of alcohol from Latvia, and Estonian alcohol taxes were decreased again as a result (for more information, see Box 5).

Although the evidence available so far implies that there is never a full substitution between recorded and unrecorded consumption, unrecorded alcohol still needs to be considered [5]. As long as unrecorded alcohol remains a cheap and available source of alcohol in a given community, it can undermine price increases and, in particular, minimum prices on recorded alcohol. The introduction of a minimum price on vodka in Russia, along with a substantial restructuring of the alcohol market and the establishment of a state enterprise distillery in 2000, did not result in a decrease in total alcohol consumption in the following years; on the contrary, there was a rise [26,66]. A steady decline in drinking levels and mortality trends was only observed starting from 2004 and 2005, when specific policies for unrecorded alcohol were introduced along with a considerable increase in alcohol taxes [30,80]. A similar scenario seems to have taken place in Belarus, where minimum prices on vodka and spirits were in place for a long time but total alcohol consumption was nevertheless increasing until 2010–2011, when penalties on home distilling were tightened, along with the introduction of other ‘best buys’ (for further details, see Box 1). At the same time, minimum prices can and were considered as a measure to reduce unrecorded alcohol use in these countries, as they give clear guidance on how much officially produced alcoholic drinks should cost; anything else below this price would be a counterfeit.

It is not possible to evaluate the effect of single measures like minimum pricing or provisions against unrecorded alcohol in the discussed countries, as they were generally introduced as part of various interventions at the same time and mostly together with increases in alcohol excise rates. But the observed trends suggest that minimum prices cannot be effective without complementary measures against unrecorded alcohol. This general implication calls into question the generalisability of the conclusion in the existing studies that, out of all existing price mechanisms, minimum unit prices would be the most effective option for reducing health inequalities [81,82]. As the available literature so far has relied solely on analyses from higher-income countries with lower shares of unrecorded alcohol among total consumption, more research based on empirical data from countries with a substantial unrecorded alcohol supply is needed to evaluate the effectiveness and cost-effectiveness of this measure in a global context.

## Conclusion

Overall, the record of policy implementations and their variability across the 15 FSU countries offers unique opportunities for studying changes in the affordability and availability of alcohol, their impact on alcohol mortality and other harms, and the important role that regulation plays in shaping these trends. The experience of the FSU countries demonstrates how drinking and mortality trends change when a state-run alcohol monopoly and rigorous alcohol control system as part of a planned economy are destroyed, and alcohol starts circulating with almost no governmental control as part of a new market economy. They are also examples of how new control measures can be implemented in response to the experienced harm from alcohol use. Thus, the highlighted experiences offer further potential insights into the complex relationship between alcohol consumption and harm at the population level, and the mediating role of socio-economic factors. Despite the declining levels of drinking in recent years, Eastern European countries still experience a much higher level of alcohol-attributable harm than one would expect from their current levels of drinking [26,32,83]. For instance, a country such as Russia—that has demonstrated a substantial gain in life expectancy due to its implementation of alcohol control measures—still has a life expectancy much lower than expected, given its relative economic wealth [27,84].

As differences in life expectancy between Western and Eastern countries of Europe are still substantially caused by alcohol, most notably by alcohol use among men in Russia, Ukraine and Belarus, followed by the three Baltic states [85], more research is needed on the differences between regions in harm per litre profiles, as well as the potential interventions that could reduce this health inequalities gap.

## Supplementary Material

dar13204-sup-0001-supinfo **Figure S1.** Political unions across the 15 Former Soviet Union countries. CIS, Commonwealth of the Independent States.
**Table S1**. Overview of the 15 Former Soviet Union countries with key indicators.
**Table S2.** Overview of national data sources that were hand-searched as part of the policy review.

## Figures and Tables

**Table 1.** Overview of main indicators of the implementation of the three ‘best buys’ policy areas (pricing, availability and marketing) across the 15 FSU countries

Country	Monopoly^a^	Minimum legal drinking age	Sale restrictions at petrol stations	Restrictions on sale hours		Minimum price	Inflation-adjusted tax^d^	Ban on special price offers promotion	Ban on promotion at point of sale	Ban on Internet and social media advertising	Ban on sponsorship of sports and youth events^c^
				Off-premises	On-premises^b^						
Armenia	No	18	No	No	No	Yes	No	No	No	No	No
Azerbaijan	No	18	No	No	No	No	No	No	N	N	Partially
Belarus	Partially	18	No	No	No	Yes	Yes	Yes	No	No	No
Estonia	No	18	Yes	Yes	No	No	No	Yes	No	No	No
Georgia	No	18	No	No	No	No	No	No	No	No	No
Kazakhstan	No	21	Yes	Yes	No	Yes	No	Yes	Yes	Yes	Yes
Kyrgyzstan	No	18	No	No	No	Yes	Yes	No	No	Yes	Partially
Latvia	No	18	No	Yes	No	No	No	No	No	No	No
Lithuania	No	20	Yes	Yes	No	No	No	Yes	No	Yes	No
Moldova	Partially	18	Yes	Yes	No	Yes	Yes	No	No	No	No
Russia	No	18	Yes	Yes	No	Yes	No	Partially	No	Yes	Partially
Tajikistan	No	18	No	No	No	No	Partially	No	Yes	Yes	Yes
Turkmenistan	Partially	18	Yes	No	No	No	No	Yes	Yes	Yes	Yes
Ukraine	No	18	No	No	No	Yes	Yes	No	No	No	No
Uzbekistan^d^	No	20	Yes	Yes	Yes	Yes	Partially	Yes	Yes	Yes	Yes

a Alcohol monopolies: Belarus and Moldova have state monopolies on production, distribution and import of alcoholic beverages, but not on retail. Turkmenistan has a state monopoly on alcohol production only. Uzbekistan had a state monopoly on the production of spirits and wine until February 2019, Ukraine had a state monopoly on the production of ethyl alcohol until January 2020. Both countries have introduced licensing regulations since then.

b Uzbekistan announced restrictions on alcohol sales in places of public catering after 9 pm in 2018.

c Tajikistan does not adjust alcohol tax for spirits and Uzbekistan does not adjust for wine.

d Belarus does not have a ban on sponsorship for youth and sports events for beer. Russia has a ban on sponsorship of sports events, but not on youth events.

**Table 2.** Overview of minimum prices for different beverage types in countries where they were introduced, in international dollars (I$) per 1 L of the named beverage for 2020 (All I$ were calculated as based on the 2019 purchasing power parities. Sources: See Table S2, Supporting Information, and in-text references)

Country (national currency ISO code)	Vodka, 1 L	Cognac, 1 L	Brandy, 1 L	Sparkling wine, 1 L	Fortified wine, 1 L	Wine, 1 L	Raw ethanol, 1 L (100%, without value-added tax)
Armenia^a^ (AMD)	15.37	15.37	15.37	4.61	6.91	4.61	–
Belarus (BYN)	18.51	–	–	–	6.11	–	10.26
Kazakhstan (KzT)	14.17	–	–	–	–	–	–
Kyrgyzstan (KGS)	13.41	13.41	13.41	-	–	–	–
Moldova (MDL)	13.74	–	–	–	–	–	–
Russia (RUB)	17.90	33.70	24.51	8.51	–	–	2.22
Ukraine^b^ (UAH)	25.22	36.20	–	17.94	9.88	8.46	–
Uzbekistan (UZS)	12.43	17.50	–	–	–	4.88	–

a Armenia: Minimum unit price is imposed on all alcoholic beverages as per Tax Code. In 2020: 6000 AMD for 1 L of 100% alcohol per beverage, introduced in 2018 as 3500 AMD for 1 L.

b Ukraine: Different minimum unit prices are imposed on spirits for 1 L of 100% alcohol per beverage type and minimum prices are imposed on wines, fortified wines and cider with a separate rate per beverage type as per the Resolution of the Cabinet of Ministers of Ukraine of 2018. Beer is the only beverage not listed and therefore without a minimum price.

## Box 1. COUNTRY SNAPSHOT: LITHUANIA—IMPLEMENTATION OF THE THREE ‘BEST BUYS’ WITHIN A SHORT PERIOD OF TIME AND THE IMMEDIATE IMPACT

With 15 L of pure alcohol consumed *per capita* per adult, **Lithuania** was one of the world’s heaviest-drinking countries in 2016 [43]. However, the most recent national data on *per capita* consumption from the Lithuanian Department of Statistics indicate a rapid decline in recorded alcohol consumption by around 1 L per year for 2017 and 2018, along with decreases in alcohol-attributable mortality rates and a general increase in life-expectancy.

For more than a decade, high levels of alcohol consumption and alcohol-related harm remained a challenge and an ongoing topic for public debate in Lithuania. The national policy response was to implement multiple ‘best buy’ policies [44] in 2008–2009, and alcohol consumption slightly decreased for the period 2008–2010, although it rose again in the following years. Since Lithuania was greatly affected in that period by the global economic crisis, it is difficult to assess the impact of these interventions. Further alcohol tax increases followed in 2014–2016, particularly a substantial tax raise in 2017, with excise taxes for wine and beer more than doubling. In 2016, a ban on alcohol sales at petrol stations was introduced, and in 2018 more ‘best buy’ policies came into effect, limiting the availability and marketing and advertising of alcohol. Also, the minimum legal drinking age was increased from 18 to 20 years and the ban on off-premise sale of alcohol at night was extended to apply between 8 pm and 10 am on Monday to Saturday nights and between 3 pm and 10 am on Sunday. A total ban on alcohol advertising on TV, radio and the Internet was introduced, with only small exceptions made for the point of sale, allowing trademark logos, for instance, to be placed on umbrellas or T-shirts [86–88]. Starting in 2020, more policy measures came into effect, banning sales from non-stationary points of sale, and cancelling temporary sale licenses, for example, for summertime, and slightly increasing the excise tax for strong beverages [89].

Preliminary analyses indicate that the introduction of these measures has caused a decline in alcohol-related mortality, and alcohol-related road traffic injuries and deaths [33,87].

## Box 2. COUNTRY SNAPSHOT: KAZAKHSTAN—A LONG-TERM APPROACH TO REDUCING ALCOHOL CONSUMPTION AND PROMOTING A HEALTHY LIFESTYLE

**Kazakhstan** presents an interesting case of a country where alcohol consumption was increasing almost steadily in the 2000s, despite the introduction of a minimum price on ethyl alcohol and vodka in 1999 [90,91] and the implementation of a total ban on alcohol advertising in 2004 [92].

However, alcohol consumption has been steadily declining since 2007 against the backdrop of stepwise increases in both alcohol excise rates and the minimum price on vodka. The Administrative Code from 2009 [93] introduced time restrictions for the off-premise sales of alcoholic beverages: sales were prohibited between 11 pm and 8 am on workdays, and between midnight and 8 am on weekends and holidays [94]. In 2010, a national development strategy paper was published by the government which set a goal of a 15% reduction in harmful alcohol use as a goal by 2020, as well as the broader goals of increasing life expectancy and forming and promoting healthy lifestyles in the population [94]. The 2014 amendments to the country’s main alcohol law [90] tightened the existing alcohol control provisions considerably. The minimum legal drinking age was raised from 18 to 21 years, and alcohol sales were prohibited in health, cultural and recreational facilities, petrol stations, during specific events and over the Internet. Moreover, the 2014 Amendments to the Administrative Code set stricter availability limitations on spirits: while alcoholic beverages with ABV of 30% and below could not be sold between 11 pm and 8 am, beverages with an ABV >30% were no longer allowed to be sold between 9 pm and 12 noon of the following day. Penalties for offenses were also increased [93].

Starting in 2016, producers of ethyl alcohol and alcoholic beverages were required to be registered within a system that would track the volume of produced alcohol and transfer the data online and in real time to a division of the Ministry of Finance [95]. This type of monitoring has proved to be essential in keeping stock of the actual volumes of alcohol; evidence from Russia, where a similar but much more advanced monitoring system was introduced in 2006, highlights that this approach has been shown to be effective in reducing the share of alcohol that avoids taxation by not being declared to be beverage alcohol [80,83].

Although there are no studies available that would systematically link the introduced policies to drinking and mortality trends, data published by the World Health Organization document a 17% decline in total alcohol consumption between 2010 and 2016, indicating that the target set by the 2010 strategy paper was achieved early on [43].

## Box 3. COUNTRY SNAPSHOT: UZBEKISTAN—FUNDAMENTAL CHANGES IN STATE ALCOHOL CONTROL AND THE ISSUE OF UNRECORDED ALCOHOL

**Uzbekistan** is one of the few countries that preserved the government’s monopoly over alcohol production, import and export over a relatively long period of time, and it introduced minimum prices on vodka and spirits as well as wine as early as 2010 [96]. The minimum prices, along with alcohol excise rates, were regularly increased over the years; in 2020, the minimum prices were more than four times higher for wine and almost six times higher for vodka and spirits than they had been in 2010—a price increase that exceeded the overall inflation rate of the national currency [97]. In 2011, the new national law ‘on limiting the availability and consumption of alcohol and tobacco products’ raised the minimum selling age for alcoholic beverages and tobacco products from 18 to 21 years and introduced mandatory health warnings at the point of sale of these products, as well as mandatory health warnings on alcohol containers with a size of at least 40% of the label [98]. Moreover, the same legislation introduced a total ban on alcohol and tobacco advertising, similar to the bans implemented in Kazakhstan and Turkmenistan. A general reduction in APC consumption has been observed since 2010–2011, although a substantial rise and fall in consumption occurred in the late 1990s and early 2000s before the introduction of these measures.

It is worth noting that despite the state monopoly on alcohol production, a considerable share of alcohol in Uzbekistan stems from unrecorded sources, with estimates of more than 50% in 2000 and about 40% in 2016 [43]. While there are no studies so far, it is possible that the tightening of licensing legislation has contributed to this slight decrease. Starting in 2007, stricter licensing procedures for alcohol retailers were introduced, and alcohol sales were banned from markets, tents and other mobile outlets [99], which are known to be common outlets of unrecorded alcohol in other former Soviet countries. Depending on the type of the issued license, different hours and time windows for off-premise sales were allowed. Also, the same legislation forbids sales at public transport stops and within a radius of 500 m from educational, sports and religious facilities; the latter provision was loosened in 2019, reducing the radius to 200 m [100]. Starting from 2016, sales of alcoholic products through vending machines that otherwise sell toys, sweets or other products aimed at children were prohibited [101]. The same legislation also explicitly forbade sales of alcoholic beverages without a written health warning or an equivalent pictogram on the label, as well as sales at outlets that lack a health warning or a sign concerning the minimum selling age. Moreover, retail sellers were from then on obliged by law to check identity documents of anyone who looks younger than 20 years of age.

In 2019, Uzbekistan abandoned its government monopoly on the production of ethyl alcohol and alcoholic beverages and introduced a licensing system instead, along with the formation of a governmental inspectorate for the regulation of the alcohol and tobacco markets under the Ministry of Finance [102]. This approach is similar to the Federal Service for Alcohol Market Regulation in Russia, a largely autonomous governmental body responsible for the regulation of excise rates and minimum prices on alcohol, and for overall surveillance of the alcohol market and alcohol tax income. Also in 2019, the government of Uzbekistan adopted a decree on a step-wise implementation of an online monitoring system that would track and record the production volume and sales of alcoholic products, following the example of the Unified State Automated Information System in Russia [83]. Currently, the system is being tested as part of a pilot project, in collaboration with a Russian company which is in charge of the implementation [103].

## Box 4. COUNTRY SNAPSHOT: BELARUS—THE EFFECTS OF AN ALCOHOL PRICING SCHEME UNDER A STATE MONOPOLY

The case of **Belarus** is a quite unique example of alcohol policy implementation, as more governmental control was kept over production and sale of alcoholic beverages. Compared to other FSU countries, Belarus has preserved a generally high level of government intervention in the economy through government ownership of various key enterprises, and a centralised planning approach with production quotas as well as price controls on many products, including alcohol. Except for retail sale, controlled through licensing, Belarus has a state monopoly on the production, distribution, import and export of ethyl alcohol and alcoholic beverages, with established annual production quotas and fixed prices [104]. Prices can be changed and adjusted for inflation by presidential decrees [105]; such pricing mechanisms have been shown to influence drinking and mortality trends [32].

In the early 1990s, alcohol availability increased substantially due to the repeal of the old Soviet monopoly and the sharp drop of alcohol prices relative to other products [56]. Moreover, traditional fruit and berry wine-making revived in the country at that time, supported by local governments. A change in legislation transferred all excise taxes on fruit wines to regional budgets, thus offering local authorities an easy and stable source of revenue [106]. A presidential decree from 1996 included ethyl alcohol and vodka in a list of goods whose prices were regulated by the government in order to ‘prevent unreasonable price increases’ in the changing market conditions [107]. In 1998, a decree of the Ministry of Economy set minimum retail prices for ethyl alcohol and vodka, and amendments of the decree in 2000 extend this regulation to alcoholic beverages with an alcohol content greater than 28% [108]. As a result, production of spirits became an important income source of revenue for the federal budget, while regional budgets became more dependent on fruit wine production. The lower production costs and excise rates made fruit wines more affordable, and their relative share of alcohol sales increased, eventually surpassing vodka. The result was that total alcohol consumption increased substantially in the early 2000s. By 2010, at 17.5 L *per capita*, Belarus had the highest level of alcohol consumption globally and, with 34.7% of all deaths being attributable to alcohol, the highest alcohol-related burden [54]. Although two national programs were launched in 2001–2005 and 2006–2010 to reduce alcohol consumption and mortality or at least prevent these indicators from rising, both campaigns failed, most likely because their measures were not based on evidence and were not planned consistently [32].

However, the most recent national program, for the period 2011–2015, appears to be a turning point. It implemented a broad portfolio of effective policies, including the WHO ‘best buys’ [44]. Alcohol excise rates were periodically increased along with minimum prices on spirits; limits were placed on alcohol retail outlets and sale hours as well as marketing; and financial penalties for drink driving and home-distilling were increased [32]. Moreover, amendments in 2016 to the presidential decree on price regulation fixed prices not only for spirits, but also for fortified fruit wines [105], and a 2017 decree of the Ministry of Antimonopoly Regulation and Trade set a minimum price for fortified fruit wines [109]. Although most of these measures are implemented at the national level, some regulations are regional. For instance, local authorities can set their own restrictions on availability of alcohol; but while some regions have strict limitations, including bans on alcohol sales at night, others have no restrictions in place or limit alcohol sales only during some special events [104].

Also, as part of the national program, the Ministry of Health made considerable efforts to improve treatment and prevention for individuals with alcohol use disorders or risk thereof, most importantly through introducing screening and brief interventions for alcohol at the level of primary health care. The latter initiative is still in its pilot phase, as the best implementation model has yet to be identified, given the gaps between primary health-care services and the specialised narcology services-a feature that many of the FSU countries share. But this appears to be a promising approach to reducing the alcohol-related burden of disease. It is worth noting that Belarus and Turkmenistan are the only two countries that have preserved the Soviet model of Occupational Therapy Rehabilitation Centres and the accompanying involuntary commitment provisions for addiction treatment.

## Box 5. COUNTRY SNAPSHOT: ESTONIA—A STEPWISE IMPLEMENTATION OF THE THREE ‘BEST BUYS’ SINCE 2008, AND SETBACKS DUE TO CROSS-BORDER ISSUES

In recent years, **Estonia** has been actively regulating the physical and economic availability of alcohol in response to the prevailing high level of alcohol consumption and alcohol-attributable harm. Analyses available to date indicate that these interventions were effective in decreasing alcohol consumption and harm, yet so far specific literature remains scarce [110–113].

Since 2008, a night ban on alcohol off-premise sales has been introduced, prohibiting sales between 10 pm and 10 am. This has contributed to a decrease in alcohol-attributable mortality, although these changes occurred against the backdrop of a financial crisis Estonia was experiencing at that time [114], which has also contributed to a general decline in drinking. The government has also pursued stringent pricing measures, increasing alcohol excise tax stepwise more than 10 times since 2005 [111]. As of January 2020, the consumer price index for alcohol and tobacco was 230% as compared to January 2005 [115]. However, the substantial price increase also had some unintended consequences, as Estonians started to engage in cross-border alcohol purchases in neighbouring Latvia [116]. This situation culminated in 2017, when alcohol tourism to Latvia tripled as compared to the previous year [111]. To counter this development, Estonia reduced its alcohol excise tax by 25% in July 2019 [117]. As a response, Latvia reduced their tax by 15% in the following month [118]. This ‘tax war’ was repeatedly criticised by public health experts and shows that there is an urgent need for harmonising alcohol policies between countries to avoid cross-border issues of unrecorded alcohol trade [119,120].

Also starting in 2008, in the new *Advertising* Act alcohol marketing was substantially limited, banning TV and radio advertisement between 7 am and 9 pm, extended in 2018 to 10 pm [121]. Moreover, in 2019 an amendment to the *Alcohol Act* introduced a partial alcohol point-of-sale display ban [122], requiring alcoholic beverages to be placed separately from other goods in stores, in a location where they would not be visible from the rest of the sales area or from outside the store. Although the policy needs to be evaluated long-term, a preliminary policy effectiveness analysis suggested that the ban decreased the estimated visibility of alcohol in stores by 15%, and that the proportion of impulse buyers of alcohol was halved [123].
