# Supplementary material for: Alcohol control policies in Former Soviet Union countries: A narrative review of three decades of policy changes and their apparent effects
Source: Drug Alcohol Rev. Author manuscript; Available in PMC 2021 Mar 7. (PMC7936953; doi:10.1111/dar.13204)
Supplement: dar13204-sup-0001-supinfo — Figure S1. Political unions across the 15 Former Soviet Union countries. CIS, Commonwealth of the Independent States. Table S1. Overview of the 15 Former Soviet Union countries with key indicators. Table S2. Overview of national data sources that were hand-searched as part of the policy review. [file NIHMS1668274-supplement-dar13204-sup-0001-supinfo.docx]

## Supporting Information


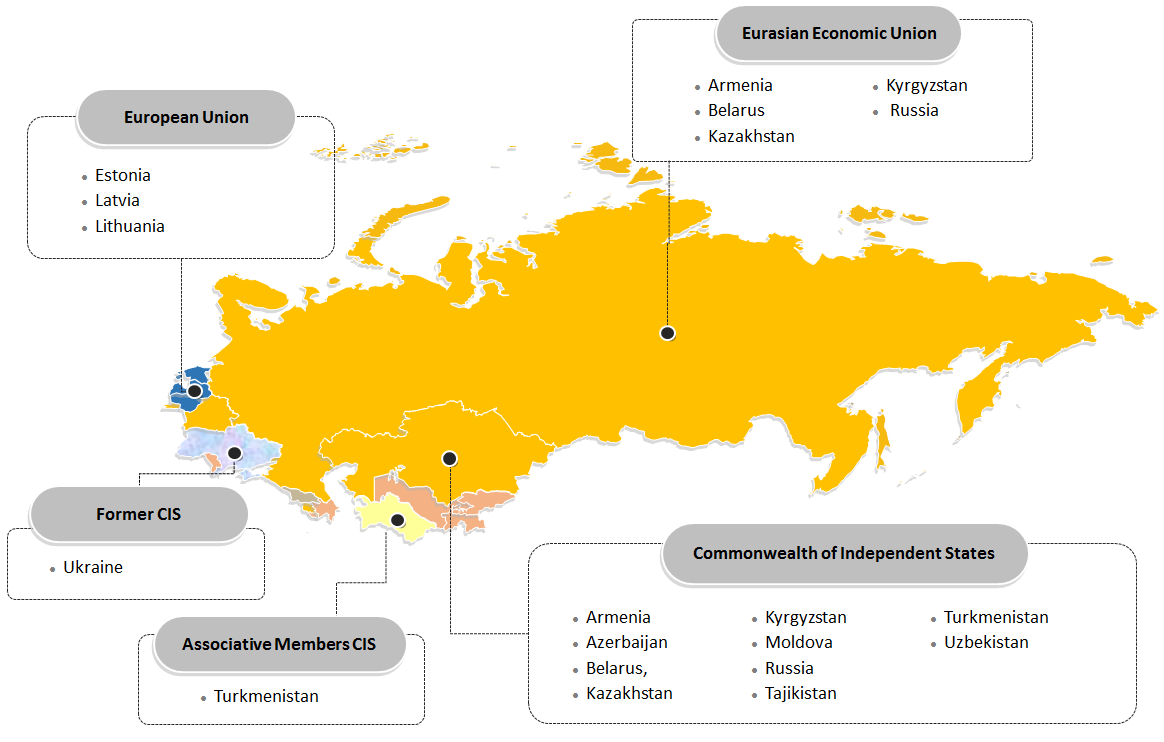


Figure S1. Political unions across the 15 Former Soviet Union countries. CIS, Commonwealth of the Independent States.

Table S1. Overview of the 15 Former Soviet Union countries with key indicators.

| Country | Region | Income level^a^ | Percentage of Muslims in population^b^ | Alcohol per capita consumption (15+) in 2010^c^ | Alcohol per capita consumption (15+) in 2018^c^ | Relative change 2010-2018 |
| --- | --- | --- | --- | --- | --- | --- |
| Armenia | Transcaucasia | UMIC | <0.1 | 5.7 | 5.5 | -2% |
| Azerbaijan | Transcaucasia | UMIC | 99.2 | 2.5 | 4.4 | +73% |
| Belarus | Eastern Europe | UMIC | <0.1 | 17.7 | 11.4 | -35% |
| Estonia | Eastern Europe | HI | 0.1 | 12.8 | 9.2 | -28% |
| Georgia | Transcaucasia | UMIC | 9.9 | 9.5 | 8.2 | -13% |
| Kazakhstan | Eastern Europe | UMIC | 56.4 | 5.0 | 4.8 | -5% |
| Kyrgyzstan | Central Asia | LMIC | 86.4 | 10.9 | 6.3 | -42% |
| Latvia | Eastern Europe | HI | <0.1 | 12.4 | 12.8 | +3% |
| Lithuania | Eastern Europe | HI | 0.1 | 15.2 | 13.2 | -13% |
| Moldova | Eastern Europe | LMIC | 0.5 | 15.5 | 11.4 | -26% |
| Russia | Eastern Europe | UMIC | 11.7 | 16.5 | 11.2 | -32% |
| Tajikistan | Central Asia | LI | 84.1 | 2.3 | 3.3 | +44% |
| Turkmenistan | Central Asia | UMIC | 93.1 | 4.4 | 4.9 | +12% |
| Ukraine | Eastern Europe | LMIC | 1.0 | 14.1 | 8.3 | -41% |
| Uzbekistan | Central Asia | LMIC | 96.3 | 3.1 | 2.6 | -16% |

**^a^** Source: World Bank, 2020. **^b^** Source: Pew Research Center, 2017. **^C^** Total alcohol per capita consumption in litres of pure alcohol, including unrecorded alcohol use, and adjusted for tourist consumption. Source: World Health Organization, 2020. HI, high-income country; LI, low-income country; LMIC, lower-middle-income country; UMIC, upper-middle-income country.

Table S2. Overview of national data sources that were hand-searched as part of the policy review**.**

| **Armenia** | Official website of the National Assembly of the Republic of Armenia:  <http://www.parliament.am/>  Legal information online database of Armenia: <https://www.arlis.am/>  National news website: https://newsarmenia.am/ |
| --- | --- |
| **Azerbaijan** | The official website of the president: <https://ru.president.az/>  State advertising agency of the Republic of Azerbaijan: <https://adra.gov.az/ru> |
| **Belarus** | Ministry of Antimonopoly Regulation and Trade: https://mart.gov.by/sites/mart/home.html//  Legal information online database: <https://www.pravo.by/>  Accounting online database: <https://www.gb.by/> |
| **Georgia** | Legal information online database: <https://matsne.gov.ge/ru> |
| **Estonia** | Legal information online database: <https://www.riigiteataja.ee/> |
| **Kazakhstan** | National information portal of the government of Kazakhstan: <https://data.egov.kz/>  Legal information online database: <https://online.zakon.kz/Lawyer> |
| **Kyrgyzstan** | Official website of the government of Kyrgyzstan: <https://www.gov.kg/ru>  Centralised data bank of legal information of the Kyrgyz Republic, Ministry of Justice:  <http://cbd.minjust.gov.kg/>  Information portal of Kyrgyzstan: <https://kginform.com/> |
| **Latvia** | Legal information online database: <https://likumi.lv/> |
| **Lithuania** | Official website of the State Tax Inspectorate Under the Ministry of Finance of the Republic of Lithuania:  <https://www.vmi.lt/cms/akcizu-istatymo-pakeitimai-nuo-2020-m.>  Lithuanian Legal Act Register: <https://www.e-tar.lt/portal/lt/index>  Official website of the Drug, Tobacco and Alcohol Control Department: <http://ntakd.lrv.lt/lt/statistika-ir-tyrimai> |
| **Moldova** | Official website of the Ministry of Justice: <https://www.legis.md/>  Governmental data portal: <https://date.gov.md/>  National news website: <https://point.md/ru/>  State Tax Service of Republic of Moldova: <https://www.sfs.md/> |
| **Russia** | Official website of the government of the Russian Federation: <http://government.ru/>  Official website of the Federal Antimonopoly Agency: <https://fas.gov.ru/>  Official website of the Federal Service for Alcohol Market Regulation:  <https://fsrar.gov.ru/>  Legal information online database: <http://consultant.ru/>  Legal information online database: <http://www.garant.ru/> |
| **Tajikistan** | National Center for Legislation under the President of the Republic of Tajikistan:  <http://ncz.tj/> |
| **Turkmenistan** | Official website of the Ministry of Justice: <http://minjust.gov.tm/ru>  National State Information Agency of Turkmenistan: <http://tdh.gov.tm/ru/>  State News Agency of Turkmenistan: <http://turkmenistan.gov.tm/> |
| **Ukraine** | Official web-portal of the Parliament of Ukraine: <https://zakon.rada.gov.ua/laws/>  Legal information online database: <https://pravo.ua/> |
| **Uzbekistan** | National database of legislation of the Republic of Uzbekistan: [https://www.lex.uz/](https://www.lex.uz/ru/)  Legal information online database: [https://regulation.gov.uz/](https://regulation.gov.uz/ru)  Governmental open data portal: <https://data.gov.uz/ru>  National News Agency Uzbekistan: <http://uza.uz/> |
